# Intimate partner violence in Nepal: Analysis of Nepal Demographic and Health Survey 2022

**DOI:** 10.1371/journal.pone.0308107

**Published:** 2024-08-16

**Authors:** Parash Mani Sapkota, Achyut Raj Pandey, Bikram Adhikari, Grishu Shrestha, Reecha Piya, Bipul Lamichhane, Shristi Garu, Deepak Joshi, Sushil Chandra Baral

**Affiliations:** 1 Research, Evaluation and Innovation Department, HERD International, Lalitpur, Nepal; 2 Manmohan Memorial Institute of Health Sciences, Kathmandu, Nepal; Washington University in St. Louis, UNITED STATES OF AMERICA

## Abstract

**Introduction:**

Intimate partner violence (IPV) is a major public health issue in Nepal. IPV has social and economic impacts on women, family, and the wider society. In this study, we aimed to determine factors associated with IPV among currently partnered women aged 15–49.

**Methods:**

We conducted a secondary data analysis of the Nepal Demographic and Health Survey (NDHS) 2022. The study examines the lifetime prevalence of IPV. IPV was measured in three domains: experience of physical violence, emotional violence, and sexual violence. Weighted univariate and multivariable logistic regression analysis were applied to determine factors associated with IPV. The results of logistic regression were presented as crude odds ratio (COR) and adjusted odds ratio (AOR) and their 95% confidence interval (CI).

**Results:**

Of 3853 women, 27.2% had experienced any form of IPV. The lifetime prevalence of physical violence, emotional violence, and sexual violence were 23.2%, 12.8%, and 7.1%, respectively. Higher odds of physical violence were reported among women aged 35–49 years (AOR: 2.13, 95% CI: 1.58–2.87), women without formal education (AOR: 1.51, 95% CI: 1.10–2.06), and women who justified wife-beating (AOR: 1.23, 95% CI: 1.00–1.52). Women from poor households (AOR: 1.61, 95% CI: 1.12–2.35) and women with uneducated partners (AOR: 1.66, 95% CI: 1.08–2.58) were at higher risk of experiencing sexual violence. Women with unemployed husbands reported a higher risk of physical violence (AOR: 2.72, 95% CI: 1.45–5.06) and emotional violence (AOR: 1.61, 95% CI: 1.12–2.35).

**Conclusion:**

Almost one in three currently partnered women experienced some form of IPV in their lifetime. Various sociodemographic, partner-related, and women’s empowerment-related factors were associated with experiencing IPV. Acknowledging and addressing these factors is essential to mitigating the high rates of IPV among reproductive aged women.

## Introduction

Violence against women and girls (VAWG) is one of the most widespread and persistent forms of human rights violations in our world today which remains unreported due to factors such as impunity, silence, stigma, and shame [1]. The UN General Assembly issued the Declaration on the Elimination of Violence Against Women in 1993 defining it as “any act of gender-based violence that results in, or is likely to result in, physical, sexual or psychological harm or suffering to women, including threats, coercion or arbitrary deprivation of liberty, whether occurring in public or in private life” [2]. The 2030 UN Agenda for Sustainable Development Goals (SDGs), calls for the elimination of all forms of violence against women and girls in both the public and private spheres, including trafficking, sexual exploitation, or other forms of exploitation in target 5.2 under goal 5 [3]. The first indicator of the target (5.2.1) specifically focuses on intimate partner violence (IPV), requiring regular reporting on “the proportion of ever-partnered women and girls aged 15 years and above subjected to physical, sexual or psychological violence by a current or former intimate partner” [3]. The World Health Organization (WHO) defines IPV as behavior by an intimate partner or ex-partner causing physical, sexual, or psychological harm. This includes physical aggression, sexual coercion, psychological abuse, and controlling behaviors [4].

Article 38 of the constitution of Nepal 2015 guarantees the protection of women against physical, mental, sexual, psychological, or any other form of violence or exploitation on any grounds and determines such acts to be punishable by law [5].Global Estimates of IPV perpetrated by men against women indicate that about one in three ever-partnered women worldwide (30%) have experienced physical and/or sexual violence at some point in time by an intimate partner [6]. Lifetime estimates of IPV prevalence range from 20% in the Western Pacific, 22% in high-income nations and Europe, and 25% in the WHO Regions of the Americas to 33% in the WHO African region, 31% in the WHO Eastern Mediterranean region, and 33% in the WHO South-East Asia region [7]. WHO estimates that 27% of ever-partnered women aged 15–49 years have experienced physical or sexual IPV or both at least once in their lifetime [8]. The estimated prevalence of lifetime IPV and past year IPV in the Southeast Asia region was 19% and 8% respectively [8].According to the NDHS 2022, about 23% of women aged 15–49 years have experienced physical violence,7% have experienced sexual violence and 13% have experienced emotional violence in their lifetime [9]. Alarmingly, about 27% of the women have experienced some form of IPV in their lifetime. There was an increase of 3% in the percentage of ever-married women who experienced IPV in the last 12 months from 14% in 2016 to 17% in 2022 [9]. Similarly, a study conducted among married women residing in the Terai region of Nepal showed that almost 16% of women reported physical violence, 18% reported sexual violence and 25% reported either or both violence perpetrated by their intimate partner [10].According to the WHO, lower levels of education, witnessing family violence, witnessing family violence, harmful use of alcohol, harmful masculine behaviors, women’s access to paid employment, and male controlling behavior towards their partners are some risk factors that contribute to the perpetration and experience of intimate partner violence [4]. Additionally, factors such as young age, low level of education, exposure to violence between parents, acceptance of violence, low economic status, and widely held beliefs about gender roles have been found to be responsible for IPV [11]. Multiple studies conducted in different settings, including Nepal have used these variables in examining their effects on IPV and its correlates [12–15]. These arrays of literature were the basis for choosing the independent variables in the study. The consequences of IPV are profound, linking with a host of different health outcomes ranging from physical injuries, bruises, unwanted pregnancies, sexually transmitted diseases, and infertility to anxiety, depression, and substance abuse [16]. In this context, this study aimed to build on the existing literature with the up-to-date findings from NDHS 2022 and contribute to the existing evidence highlighting the association of individual and husband characteristics with IPV.

## Methods

### Study design

We performed a secondary analysis of data from the NDHS 2022 which is the sixth nationally representative survey of its kind implemented by New ERA, under the aegis of the Ministry of Health and Population of Nepal with technical support from ICF International and financial support from the United States Agency for International Development (USAID) [9]. The dataset was publicly available on the official website of ‘The DHS program’ [17]. The details of the questionnaire and study methodology have been described in the NDHS 2022 report [9].

### Sample size and sampling technique

The details about sample size and sampling technique are explained in detail in NDHS 2022 report [9]. In brief, a total of 14,845 women aged 15–49 were successfully interviewed, yielding a response rate of 97%. A total of 5,177 women irrespective of their relationship status were selected and interviewed for the domestic violence module. Special weights were used to adjust for the selection of only one woman per household and to ensure the subsample was representative nationally. Only seven women were selected for the module but were not interviewed with the Woman’s Questionnaire, and six who were selected and interviewed with the Woman’s Questionnaire could not complete the module due to privacy concerns. Partner/Husband-related information was captured only for women currently in an intimate relationship hence we selected 4211 women (unweighted number) who are currently in an intimate relationship out of 5,177 total women selected for the domestic violence module. This allowed us to analyze the association of partner characteristics with IPV.

### Data collection tool

The 2022 NDHS survey administered four major questionnaires: the Household Questionnaire, the Woman’s Questionnaire, the Man’s Questionnaire, and the Biomarker Questionnaire. The Woman’s Questionnaire was used to collect information on domestic violence from women aged 15–49 years.

The module on domestic violence was confined to respondents selected for the domestic violence module from the subsample of households selected for the men’s survey such that only one eligible woman per household was selected. The module was implemented only when privacy could be maintained, and the information collection process followed the WHO-recommended guidelines and ethical standard [18].

More recently, the questionnaire module used to capture IPV in NDHS 2022 survey was revised to also capture IPV experienced by never married women who reported that they currently or formerly had an intimate partner. In the context of the revised questionnaire module and this report, the term “boyfriend” excludes anyone reported as an intimate partner.

### Measurement of variables

#### Outcome variables

The outcome variables for this study were lifetime experience of physical violence, lifetime experience of emotional violence, lifetime experience of sexual violence and lifetime experience of any form of violence perpetrated by the current partners among women aged 15–49 years currently in an intimate relationship. The outcome variable was measured by self-reported experiences of the women. We adopted the operational definition of three forms of violence along with any form of IPV, as stated in the original report [9] which is presented in Table 1.

**Table 1:** Measure of different types of violence.

Forms of Violence	Operational definitions
Physical violence	The women were considered to have experienced physical violence if their husband or partner ever did the following a. Push, shake, or throw something at her b. Slap her c. Twist her arm or pull her hair d. Punch her with a fist or something that could hurt e. Kick, drag, or beat her f. Try to choke her or burn her on purpose g. Attack her with a knife, gun, or other weapon
Emotional violence	The women were considered to have experienced emotional violence if their husband or partner ever did the following h. Said or did something to humiliate her in front of others i. Threatened to hurt or harm her or someone she cared about j. Insulted to make her feel bad about herself
Sexual violence	The women were considered to have experienced sexual violence if their husband or partner ever did the following k. Physically force her to have sexual intercourse l. Physically force her to perform any other sexual acts m. Force her with threats or in any other way to perform sexual acts
Any form of IPV	The women were considered to have experienced any form of IPV if they experienced physical violence and/or emotional violence and/or sexual violence

Each question was summarized into binary responses ‘Yes/No’ to capture the lifetime experience of different forms of IPV. A value of 1 was given if the event took place (Often, Sometimes, yes but not in the last 12 months) and a value of 0 was given if the act did not take place. For physical violence, the aggregate of a to g was calculated; the woman was considered ‘experiencing physical violence’ if the aggregate was 1 or more. Using similar logic, the aggregate of h to j was used for categorizing ‘experiencing emotional violence’ and ‘not experiencing emotional violence’ and the aggregate of k to m was used for categorizing ‘experiencing sexual violence’ and ‘not experiencing sexual violence.’ Similarly, lifetime experience of any form of IPV was categorized as ‘experiencing any form of violence’ if the respondent experienced physical and/or emotional and/or sexual violence and categorized as ‘not experiencing any form of violence’ if the respondent didn’t experience physical, emotional and sexual violence.

#### Independent variables

In this study, independent variables included socio-economic variables, partner-related characteristics, and women empowerment-related variables similar to a previous study [13]. The socio-economic variables were the age of women (in years), ethnicity (Brahmin or Chettri/ Dalits/ Janajatis/ Muslim/ Other castes), province (Koshi/ Madhesh/ Bagmati/ Gandaki/ Lumbini/ Karnali/ Sudurpashchim), type of residence (rural/urban), wealth index (Rich/ Middle/ Poor),, and witnessing parental violence (yes/ no).. Partner-related characteristics were the partner’s education, partner’s occupation, partner’s alcohol consumption, control behavior displayed, and the respondent being afraid of their partner. The women empowerment-related variables included education, occupation, exposure to the internet, exposure to media, ownership of property, participation in household decisions, attitude towards autonomy of sexual rights, and attitude towards justification of beating by partner. [S1 File].

### Statistical analysis

We used R version 4.2.0 [19] and RStudio [20] for pre-analytical processing and statistical analysis. We performed a weighted analysis to account for the complex survey design of NDHS 2022 and non-response rates using the “survey” package [21]. We presented categorical variables as frequency, percentage (%), and 95% confidence interval (CI) whereas numerical variables as mean and standard deviation. We carried out univariate and multivariable logistic regression using “stats” [19] package to determine factors associated with IPV. The results of the logistic regression were presented as crude odds ratio (COR) and adjusted odds ratio (AOR) and their 95% CI using “gtsummary” package [22]. We included all the variables with p < 0.2 in univariate logistic regression in the multivariable regression model [23]. No collinearity was found between variables when checked for Variance Inflation Factor for regression models (Physical Violence model (Mean: 1.61, Range: 1.03–2.95), Emotional Violence model (Mean: 1.65, Range: 1.06–3.15), Sexual Violence model (Mean: 1.68, Range: 1.07–3.34), Physical Violence model (Mean: 1.65, Range: 1.06–3.15)) using the performance package [24]. A p-value of <0.05 was considered statistically significant.

### Ethical approval

We obtained permission for the NDHS 2022 dataset from “The DHS program” upon registration and providing the research title and research purpose. NDHS 2022 received ethical approval for the survey from the Ethical Review Board of Nepal Health Research Council (Reference number: 494–2021) and the institutional review board of ICF International (Reference number: 180657.0.001.NP.DHS.01, 28^th^ April 2022). In NDHS 2022, written informed consent was obtained from every participant before enrolling them in the study.

## Results

### Characteristics of the study population

Table 2 shows various socio-demographic characteristics of the study participants. Of the total 3,853 women (weighted number), only 0.4% were living together and the remaining 99.6% were married. The age of the respondents ranged between 15 to 49 years with the highest number of respondents in the age group between 35 to 49 years (41.2%). Most of the respondents were from Janajati among the prevalent castes, accounting for 36.8%. In terms of provinces, Madhesh (22%), Bagmati (18.4%), Lumbini (17.7%), and Koshi (16.9%) are among the most highly represented provinces. Two-thirds of the respondents (66.6%) were from urban areas. The majority of the participants were from the richest wealth quintile (40.8%). The experience of witnessing parental violence in the form of father beating the mother was low with 16.2% of them reporting it.

**Table 2:** Socio-demographic characteristics of the study population.

	n = 3,853		
Characteristics	Frequency^#^	%^#^	95% CI
**Marital Status**			
Living Together	14	0.4	0.2, 0.8
Married	3839	99.6	99.2, 99.8
**Age (in years)**			
15–24	814	21.10	19.6, 22.8
25–34	1453	37.7	35.9, 39.5
35–49	1586	41.2	39.3, 43.0
Mean age (SD)	32.4 (8.6)		32, 33
**Ethnicity**			
Brahmin/Chettri	1072	27.8	26.2, 29.5
Dalits	581	15.1	13.8, 16.4
Janajatis	1419	36.8	35.0, 38.7
Muslim	174	4.5	3.7, 5.5
Other castes	607	15.8	14.3, 17.3
**Province**			
Bagmati province	707	18.4	16.8, 20.0
Gandaki province	371	9.6	8.7, 10.7
Karnali province	253	6.6	5.9, 7.3
Koshi province	650	16.9	15.5, 18.3
Lumbini province	682	17.7	16.3, 19.2
Madhesh province	848	22.0	20.4, 23.7
Sudurpashchim province	343	8.9	8.1, 9.8
**Residence**			
Urban	2568	66.6	65.0, 68.2
Rural	1285	33.4	31.8, 35.0
**Wealth Index**			
Rich	1572	40.8	38.9, 42.7
Middle	796	20.7	19.2, 22.2
Poor	1485	38.5	36.8, 40.3
**Witnessed Parental Violence**			
No	3230	83.8	82.4, 85.1
Yes	623	16.2	14.9, 17.6

*# weighted; %*: *Percentage; CI*: *Confidence interval; SD*: *Standard deviation*.

### Husband/ Partner characteristics of the study population

Table 3 shows the characteristics of the respondent’s partner. About 44.2% of the respondent’s partners had received a secondary or higher level of education followed by a basic level of education (40%) and no education or unknown (15.8%). The majority of the respondent’s partners were engaged in manual work (44.8%) followed by clerical or sales (23.9%), agriculture (18.8%), professional or technical or managerial work (10.2%) and 2.2% did not have any work. Of the total respondents, 64.9% of the respondents reported that their partner did not drink alcohol or were never drunk, and 35.1% reported that they were sometimes drunk or often drunk. About two-thirds of the respondents (66.8%) reported that their partners did not exhibit any controlling behavior and 33.2% had exhibited controlling behavior. Of the total respondents, 43.8% were never afraid of their partner and 56.2% were sometimes or most of the time afraid of their partner.

**Table 3:** Husband/Partner characteristics of study population.

	n = 3,853		
Characteristics	Frequency^#^	%^#^	95% CI
**Husband/Partner’s Education**			
Secondary or Higher	1703	44.2	42.3, 46.1
Basic	1539	40.0	38.1, 41.8
No education	610	15.8	14.5, 17.3
**Husband/Partner’s Occupation**			
Professional/technical/managerial	394	10.2	9.1, 11.4
Agriculture	725	18.8	17.5, 20.2
Clerical/Sales/Others	922	23.9	22.3, 25.6
Manual Work	1727	44.8	43.0, 46.7
Not working	86	2.2	1.7, 2.8
**Husband/Partner’s Alcohol consumption**			
Does not drink/Never drunk	2501	64.9	63.1, 66.7
Is sometimes/often drunk	1352	35.1	33.3, 36.9
**Control Behavior Displayed by Husband/Partner**			
No behavior displayed	2574	66.8	65.0, 68.5
Control behavior displayed	1279	33.2	31.5, 35.0
**Respondent Afraid of Partner**			
Never Afraid	1689	43.8	42.0, 45.7
Sometimes or Most of the time afraid	2164	56.2	54.3, 58.0

# *weighted*; %: *Percentage*; *CI*: *Confidence interval*.

### Empowerment characteristics of the study population

Table 4 shows the empowerment characteristics of women in the study. The study revealed that most of the respondents (34.7%) had received a secondary or higher level of education. There was a diverse range of occupational distribution among the respondents with the majority (53.6%) engaged in agriculture. A substantial majority of the respondents used the internet (61.6%), were exposed to media (78.2%), and did not own any property (81.5%). The majority (83.7%) of respondents reported participation in household decision-making, more than three quarters (77.3%) of respondents reported having the autonomy of sexual rights and 80.6% of respondents agreed that wife beating is not justified.

**Table 4:** Empowerment characteristics of the study population.

	n = 3,853		
Characteristics	Frequency^#^	%^#^	95% CI
**Education**			
Secondary or Higher	1336	34.7	32.9, 36.5
Basic	1293	33.6	31.8, 35.3
No education	1224	31.8	30.0, 33.6
**Occupation**			
Professional/technical/managerial	198	5.1	4.3, 6.1
Agriculture	2065	53.6	51.7, 55.5
Clerical/Sales/Others	360	9.3	8.2, 10.6
Manual Work	316	8.2	7.2, 9.3
Not working	914	23.7	22.0, 25.5
**Internet Exposure**			
No	1481	38.4	36.7, 40.2
Yes	2372	61.6	59.8, 63.3
**Media Exposure**			
Exposure to media	3014	78.2	76.6, 79.7
No exposure	839	21.8	20.3, 23.4
**Ownership of Property**			
Does not own at all	3139	81.5	79.9, 82.9
Owns either alone or jointly	715	18.5	17.1, 20.1
**Participation in household decision-making**			
Participation	3227	83.7	82.2, 85.2
No participation	627	16.3	14.8, 17.8
**Attitude toward autonomy of sexual rights**			
Accepts sexual right	2977	77.3	75.6, 78.8
Does not accept sexual rights	877	22.7	21.2, 24.4
**Attitude toward justification for beating wife**			
Not justified	3107	80.6	79.1, 82.1
Justified for one or more reasons	746	19.4	17.9, 20.9

*# weighted; %*: *Percentage; CI*: *Confidence interval*.

### Prevalence of different forms of IPV

Table 5 shows the prevalence of lifetime experience of IPV. Overall, 27.2% of the respondents experienced at least one type of violence from their current husbands or partners in their lifetime, with physical violence being the most common (23.2%). It was followed by emotional violence (12.8%) and sexual violence (7.1%).

**Table 5:** Experience of different forms of IPV.

Forms of violence (lifetime)	n = 3,853		
	Frequency^#^	%^#^	95% CI
Emotional violence	492	12.8	11.5, 14.1
Physical violence	895	23.2	21.7, 24.9
Sexual violence	272	7.1	6.2, 8.1
Any form of IPV	1,047	27.2	25.5, 28.9

*# weighted; %*: *Percentage; CI*: *Confidence interval*.

### Bivariate analysis of IPVs by background characteristics

Table 6 shows the lifetime experience of physical, emotional, and sexual violence by explanatory variables. The women of age group 35–49 years had high proportions of physical (24.6%) and sexual (7.7%) violence whereas, women aged 15–24 years had high proportions of emotional violence (13%). The proportion of lifetime experience of all three violence namely physical, emotional and sexual violence was higher among the respondents: of Muslim ethnic group (49.3%, 27.5% and 16.3%); Madhesh province (39%, 24.9% and 11.9%); rural place of residence (23.4%, 13.2% and 7.4%); poor wealth index (27.3%, 14.5% and 10%); those witnessing parental violence (41.4%, 23.1% and 14.8%); whose husband/ partner had no education (35.8%, 22.8% and 12.6%); whose husband/ partner did not have any work (38.8%, 29.4% and 11.5%); whose husband/ partner was sometimes or often drunk (37.7%, 21.8% and 13.1%); whose husband/ partner displayed one or more controlling behavior (44.9%, 30.2% and 17.7%); who were afraid of their husband/partner sometimes or most of the times (32.2%, 18.2% and 11%); who had no education (33.4%, 18.3% and 9.9%); who were involved in manual work (31.7%, 16.5% and 10.4%); who didn’t have exposure to internet (27.2%, 14.3% and 9%); who didn’t have exposure to any media (32.3%, 23.3% and 10.1%); who had no ownership of property (23.9%, 12.9% and 7.1%); who had no participation in household decision making (27.3%, 20.3% and 10%); who didn’t believe in autonomy of sexual rights (35.4%, 21.6% and 12.5%) and respondents who agreed that wife beating is justified for one or more reasons (28.2%, 13.7% and 8.1%). Similarly, a higher proportion of women from the Muslim community (55.5%) and Madhesh province (45.8%) experienced either form of IPV. The proportion was also high for women who experienced parental violence (45.7%). The proportion was specifically high in women whose partners were not educated (42.5%), not working (44%), and were drunk sometimes or often (42.6%).

**Table 6:** Experience of different IPVs by background characteristics.

Characteristics	Physical Violence (n = 895)			Emotional Violence (n = 492)			Sexual Violence (n = 272)			Any form of IPV (n = 1047)		
	n^*1*^	% (95% CI)^*2*^	p-value^*3*^	n^*1*^	% (95% CI)^*2*^	p-value^*3*^	n^*1*^	% (95% CI)^*2*^	p-value^*3*^	n^*1*^	% (95% CI)^*2*^	p-value^*3*^
**Age**			0.059			0.978			0.449			0.567
15–24	158	19.4 (16.0, 23.3)		106	13.0 (10.2, 16.4)		59	7.3 (5.2, 10.0)		207	25.4 (21.6, 29.5)	
25–34	346	23.8 (21.5, 26.4)		183	12.6 (10.8, 14.6)		91	6.3 (5.1, 7.8)		402	27.7 (25.2, 30.3)	
35–49	391	24.6 (22.2, 27.3)		203	12.8 (10.9, 15.0)		122	7.7 (6.2, 9.4)		438	27.6 (25.1, 30.3)	
**Ethnicity**			**<0.001**			**<0.001**			**<0.001**			**<0.001**
Brahmin/Chettri	150	14.0 (11.8, 16.5)		87	8.1 (6.46, 10.1)		47	4.4 (3.3, 5.9)		179	16.7 (14.4, 19.3)	
Dalits	188	32.4 (28.2, 36.9)		93	16.1 (13.1, 19.6)		54	9.4 (7.2, 12.1)		209	36.0 (31.7, 40.5)	
Janajatis	248	17.5 (15.3, 19.9)		120	8.5 (6.89, 10.4)		78	5.5 (4.2, 7.1)		292	20.6 (18.2, 23.1)	
Muslim	86	49.3 (39.3, 59.3)		48	27.5 (19.4, 37.4)		28	16.3 (10.1, 25.4)		97	55.5 (45.4, 65.2)	
Other Castes	223	36.7 (31.8, 41.8)		144	23.6 (19.5, 28.4)		64	10.6 (7.7, 14.3)		270	44.5 (39.3, 49.8)	
**Province**			**<0.001**			**<0.001**			**<0.001**			**<0.001**
Bagmati province	110	15.6 (12.3, 19.5)		59	8.3 (5.99, 11.4)		39	5.5 (3.7, 8.2)		128	18.1 (14.6, 22.3)	
Gandaki province	56	15.1 (11.9, 19.2)		32	8.6 (6.15, 11.9)		17	4.5 (3.0, 6.8)		71	19.0 (15.3, 23.4)	
Karnali province	45	17.6 (13.8, 22.2)		30	11.8 (8.42, 16.4)		22	8.9 (6.5, 11.9)		58	23.0 (18.9, 27.8)	
Koshi province	136	20.9 (17.4, 24.8)		60	9.2 (6.84, 12.2)		43	6.5 (4.5, 9.4)		153	23.6 (20.0, 27.6)	
Lumbini province	158	23.2 (19.9, 26.9)		76	11.1 (8.78, 14.0)		34	5.0 (3.6, 7.0)		179	26.3 (22.8, 30.1)	
Madhesh province	331	39.0 (34.8, 43.4)		211	24.9 (21.3, 29.0)		101	11.9 (9.3, 15.2)		388	45.8 (41.4, 50.2)	
Sudurpashchim province	60	17.4 (13.9, 21.5)		24	7.1 (5.14, 9.83)		16	4.6 (2.9, 7.3)		69	20.3 (16.6, 24.5)	
**Residence**			0.846			0.626			0.585			0.484
Urban	594	23.1 (21.1, 25.3)		323	12.6 (11.0, 14.3)		177	6.9 (5.7, 8.3)		688	26.8 (24.6, 29.1)	
Rural	301	23.4 (21.3, 25.7)		169	13.2 (11.5, 15.1)		95	7.4 (6.2, 8.8)		359	27.9 (25.7, 30.3)	
**Wealth Index**			**<0.001**			**0.011**			**<0.001**			**<0.001**
Rich	290	18.5 (16.1, 21.1)		163	10.4 (8.51, 12.6)		72	4.6 (3.4, 6.2)		336	21.4 (18.9, 24.2)	
Middle	199	25.0 (21.6, 28.8)		114	14.3 (11.7, 17.4)		51	6.4 (4.7, 8.7)		241	30.3 (26.5, 34.2)	
Poor	406	27.3 (24.9, 29.9)		215	14.5 (12.6, 16.5)		149	10.0 (8.4, 11.9)		470	31.6 (29.2, 34.2)	
**Parental Violence**			**<0.001**			**<0.001**			**<0.001**			**<0.001**
Did not experience	637	19.7 (18.1, 21.4)		348	10.8 (9.55, 12.1)		180	5.6 (4.7, 6.6)		762	23.6 (21.9, 25.4)	
Experienced	258	41.4 (36.9, 46.0)		144	23.1 (19.3, 27.4)		92	14.8 (11.7, 18.6)		285	45.7 (41.1, 50.3)	
**Husband/Partner’s Education**			**<0.001**			**<0.001**			**<0.001**			**<0.001**
Secondary or Higher	254	14.9 (13.0, 17.0)		147	8.6 (7.19, 10.4)		60	3.5 (2.7, 4.7)		309	18.2 (16.1, 20.4)	
Basic	423	27.5 (24.9, 30.2)		205	13.3 (11.4, 15.5)		136	8.8 (7.3, 10.7)		479	31.1 (28.4, 33.9)	
No education	219	35.8 (31.2, 40.6)		139	22.8 (18.9, 27.4)		77	12.6 (9.6, 16.3)		259	42.5 (37.7, 47.4)	
**Husband/Partner’s Occupation**			**<0.001**			**<0.001**			**0.013**			**<0.001**
Professional/technical/managerial	50	12.7 (9.40, 17.0)		28	7.0 (4.67, 10.4)		16	4.1 (2.3, 7.2)		66	16.6 (12.8, 21.3)	
Agriculture	148	20.5 (17.3, 24.0)		84	11.5 (9.07, 14.6)		57	7.8 (5.9, 10.4)		176	24.3 (20.9, 27.9)	
Clerical/Sales/Others	204	22.1 (18.9, 25.7)		98	10.6 (8.37, 13.3)		46	5.0 (3.4, 7.4)		232	25.2 (21.8, 28.9)	
Manual	460	26.6 (24.2, 29.2)		258	14.9 (13.0, 17.1)		143	8.3 (6.9, 9.9)		536	31.0 (28.5, 33.7)	
Not working	33	38.8 (27.4, 51.7)		25	29.4 (19.2, 42.3)		10	11.5 (6.1, 20.9)		38	44.0 (32.1, 56.5)	
**Husband/Partner’s Alcohol consumption**			**<0.001**			**<0.001**			**<0.001**			**<0.001**
Does not drink/Never Drunk	385	15.4 (13.7, 17.2)		196	7.9 (6.65, 9.26)		95	3.8 (3.1, 4.7)		471	18.8 (17.1, 20.8)	
Is sometimes/often Drunk	510	37.7 (34.7, 40.8)		295	21.8 (19.3, 24.6)		177	13.1 (11.0, 15.5)		576	42.6 (39.5, 45.7)	
**Control Behavior Displayed by Husband/Partner**			**<0.001**			**<0.001**			**<0.001**			**<0.001**
No behavior displayed	320	12.4 (11.0, 14.1)		105	4.1 (3.18, 5.23)		46	1.8 (1.3, 2.5)		373	14.5 (12.9, 16.2)	
One or more Control Behavior Displayed	575	44.9 (41.7, 48.2)		387	30.2 (27.3, 33.3)		226	17.7 (15.3, 20.4)		674	52.7 (49.4, 55.9)	
**Respondent Afraid of Partner**			**<0.001**			**<0.001**			**<0.001**			**<0.001**
Never Afraid	199	11.8 (10.2, 13.6)		97	5.8 (4.58, 7.21)		34	2.0 (1.5, 2.8)		249	14.7 (12.9, 16.8)	
Sometimes or Most of the time afraid	696	32.2 (29.8, 34.6)		395	18.2 (16.3, 20.3)		238	11.0 (9.5, 12.7)		798	36.9 (34.5, 39.4)	
**Education**			**<0.001**			**<0.001**			**<0.001**			**<0.001**
Secondary or Higher	173	13.0 (11.0, 15.3)		110	8.3 (6.73, 10.1)		45	3.3 (2.4, 4.6)		216	16.2 (14.0, 18.6)	
Basic	312	24.1 (21.5, 27.0)		157	12.1 (10.2, 14.3)		106	8.2 (6.6, 10.1)		359	27.8 (25.0, 30.7)	
No education	409	33.4 (30.3, 36.7)		224	18.3 (15.7, 21.3)		121	9.9 (8.0, 12.2)		472	38.5 (35.3, 41.9)	
**Occupation**			**<0.001**			**0.024**			0.125			**<0.001**
Professional/technical/managerial	24	11.9 (7.61, 18.2)		12	5.9 (3.09, 10.9)		10	5.0 (2.4, 10.1)		30	15.3 (10.4, 22.1)	
Agriculture	514	24.9 (22.9, 27.0)		278	13.4 (11.9, 15.2)		157	7.6 (6.5, 8.9)		595	28.8 (26.7, 31.0)	
Clerical/Sales/Others	55	15.3 (11.1, 20.7)		33	9.1 (5.98, 13.7)		17	4.6 (2.3, 9.0)		66	18.2 (13.7, 23.8)	
Manual	100	31.7 (25.9, 38.1)		52	16.5 (12.0, 22.2)		33	10.4 (6.8, 15.6)		112	35.4 (29.3, 42.1)	
Not working	202	22.1 (18.7, 26.0)		117	12.9 (10.1, 16.2)		56	6.1 (4.3, 8.6)		244	26.7 (23.0, 30.7)	
**Internet Exposure**			**<0.001**			0.061			**0.002**			**<0.001**
No	402	27.2 (24.6, 29.9)		212	14.3 (12.3, 16.5)		133	9.0 (7.4, 10.8)		459	31.0 (28.4, 33.8)	
Yes	493	20.8 (18.8, 22.8)		280	11.8 (10.3, 13.5)		139	5.9 (4.8, 7.2)		588	24.8 (22.7, 27.0)	
**Media Exposure**			**<0.001**			**<0.001**			**0.001**			**<0.001**
Yes	624	20.7 (19.0, 22.5)		297	9.8 (8.66, 11.2)		188	6.2 (5.3, 7.3)		719	23.8 (22.1, 25.7)	
No	271	32.3 (28.6, 36.3)		195	23.3 (19.9, 27.0)		85	10.1 (7.9, 12.8)		328	39.1 (35.2, 43.2)	
**Ownership of Property**			0.075			0.683			0.920			**0.040**
Does not own at all	751	23.9 (22.2, 25.7)		405	12.9 (11.5, 14.4)		222	7.1 (6.1, 8.3)		879	28.0 (26.2, 29.9)	
Owns either alone or jointly	144	20.2 (16.9, 23.9)		87	12.2 (9.63, 15.4)		50	7.0 (5.1, 9.5)		168	23.5 (20.0, 27.4)	
**Participation in household decision-making**			**0.033**			**<0.001**			**0.013**			**<0.001**
Participation	724	22.4 (20.8, 24.2)		364	11.3 (10.1, 12.7)		210	6.5 (5.6, 7.7)		833	25.8 (24.1, 27.6)	
No participation	171	27.3 (23.2, 32.0)		127	20.3 (16.5, 24.7)		63	10.0 (7.4, 13.4)		214	34.2 (29.6, 39.1)	
**Attitude toward autonomy of sexual rights**			**<0.001**			**<0.001**			**<0.001**			**<0.001**
Accepts sexual right	585	19.7 (18.0, 21.4)		303	10.2 (8.94, 11.5)		162	5.5 (4.5, 6.6)		679	22.8 (21.1, 24.7)	
Does not accept sexual rights	310	35.4 (31.7, 39.2)		189	21.6 (18.4, 25.1)		110	12.5 (10.2, 15.3)		368	42.0 (38.1, 46.0)	
**Attitude toward justification for beating wife**			**0.002**			0.500			0.310			**0.004**
Not justified	684	22.0 (20.3, 23.8)		390	12.5 (11.2, 14.0)		212	6.8 (5.8, 7.9)		807	26.0 (24.2, 27.9)	
Justified for one or more reasons	211	28.2 (24.6, 32.2)		102	13.7 (10.9, 17.0)		61	8.1 (6.0, 10.9)		240	32.2 (28.4, 36.3)	

^*1*^ n (%).

^*2*^ CI = Confidence Interval.

^*3*^ chi-squared test with Rao & Scott’s second-order correction.

Chi-square analysis indicated that respondents who experienced all forms of violence had significant association with explanatory variables such as; ethnicity, province, wealth index, witnessing parental violence, husband/ partner’s education, husband/ partner’s occupation, husband/ partner’s alcohol use, husband/ partners with controlling behavior, respondents afraid of their husband/ partners, respondents education, exposure to media, respondents participation in household decision making and respondents attitude towards the autonomy of sexual rights. The type of women’s occupation was significantly associated with both physical and emotional violence. Women’s exposure to the internet was significantly associated with physical and sexual violence whereas, women’s attitude towards wife beating was significantly associated with physical violence. Any form of IPV was associated with ethnicity, province, wealth, education, occupation, media exposure, internet exposure, ownership of property, participation in household decision-making, attitude towards autonomy of sexual rights, attitude towards justification for beating wife, experience of parental violence and husband/partner related characteristics**.**

### Factors associated with lifetime experience of different forms of IPV with current partner

The logistic regression analysis of the factors associated with lifetime experience of different forms of IPVs is shown in Table 7. The multivariate regression model showed that women from the Muslim community were more than three times more likely to experience either form of IPV (AOR: 3.17, CI: 2.01–5.04) than women from the Brahmin/Chettri community. Women who experienced parental violence were twice as likely (AOR: 2.09, CI: 1.69–2.58) to experience IPV than those who did not. Women whose partners were not working were more than two and a half times more likely (AOR: 2.58, CI: 1.40–4.73) to experience IPV than those whose partners were engaged in professional/technical/managerial positions. Similarly, women whose partners were sometimes or often drunk had higher odds of experiencing IPV (AOR: 2.73, CI: 2.29–3.25) than those whose partners did not drink or were never drunk.

**Table 7:** Analysis of factors associated with lifetime experience of different forms of IPVs with current partner among women in Nepal, 2022 NDHS.

Characteristics	Physical Violence		Emotional Violence		Sexual Violence		Any form of IPV	
	Crude OR	Adjusted OR	Crude OR	Adjusted OR	Crude OR	Adjusted OR (95% CI)	Crude OR	Adjusted OR
	(95% CI)	(95% CI)	(95% CI)	(95% CI)	(95% CI)		(95% CI)	(95% CI)
**Age group in years**								
15–24	Ref	Ref	Ref		Ref		Ref	
25–34	1.30 (0.99, 1.70)	1.76 (1.37, 2.29) **	0.97 (0.70, 1.33)		0.85 (0.56, 1.30)		1.13 (0.88, 1.44)	
35–49	1.36 (1.04, 1.78) *	2.13 (1.58, 2.87) **	0.98 (0.71, 1.36)		1.06 (0.70, 1.61)		1.12 (0.88, 1.44)	
**Ethnicity**								
Brahmin/Chettri	Ref	Ref	Ref	Ref	Ref	Ref	Ref	Ref
Dalits	2.95 (2.24, 3.89) **	1.50 (1.11, 2.02) **	2.18 (1.55, 3.07) **	0.89 (0.61, 1.30)	2.23 (1.47, 3.39) **	0.85 (0.53, 1.35)	2.80 (2.16, 3.64) **	1.24 (0.93, 1.65)
Janajatis	1.31 (1.02, 1.68) *	0.81 (0.63, 1.04)	1.05 (0.76, 1.46)	0.70 (0.50, 0.97) *	1.25 (0.83, 1.89)	0.76 (0.51, 1.14)	1.29 (1.02, 1.62) *	0.78 (0.62, 1.00) *
Muslim	5.98 (3.82, 9.36) **	3.64 (2.29, 5.82) **	4.31 (2.58, 7.20) **	1.41 (0.81, 2.43)	4.22 (2.24, 7.96) **	1.98 (1.03, 3.79) *	6.22 (3.99, 9.69) **	3.17 (2.01, 5.04) **
Other Castes	3.57 (2.67, 4.76) **	2.07 (1.48, 2.90) **	3.52 (2.49, 4.97) **	1.68 (1.11, 2.54) *	2.56 (1.61, 4.05) **	1.25 (0.74, 2.13)	4.00 (3.04, 5.27) **	2.35 (1.70, 3.26) **
**Province**								
Bagmati province	Ref	Ref	Ref	Ref	Ref	Ref	Ref	Ref
Gandaki province	0.97 (0.65, 1.43)	0.90 (0.61, 1.31)	1.04 (0.63, 1.72)	0.89 (0.55, 1.43)	0.81 (0.44, 1.49)	0.69 (0.37, 1.25)	1.06 (0.73, 1.53)	0.99 (0.69, 1.41)
Karnali province	1.16 (0.78, 1.72)	0.73 (0.46, 1.13)	1.49 (0.89, 2.49)	0.88 (0.52, 1.48)	1.66 (0.97, 2.84)	0.87 (0.47, 1.58)	1.35 (0.94, 1.94)	0.87 (0.57, 1.31)
Koshi province	1.43 (1.00, 2.03) *	1.00 (0.73, 1.37)	1.12 (0.70, 1.79)	0.68 (0.45, 1.03)	1.20 (0.67, 2.13)	0.58 (0.36, 0.93) *	1.39 (1.00, 1.94)	0.93 (0.69, 1.26)
Lumbini province	1.63 (1.17, 2.28) **	1.12 (0.82, 1.52)	1.39 (0.89, 2.15)	0.80 (0.54, 1.19)	0.90 (0.52, 1.56)	0.47 (0.28, 0.77) **	1.61 (1.17, 2.22) **	1.05 (0.78, 1.41)
Madhesh province	3.46 (2.50, 4.80) **	1.40 (0.99, 1.99)	3.68 (2.45, 5.52) **	1.02 (0.66, 1.57)	2.32 (1.39, 3.84) **	0.59 (0.35, 1.00) *	3.81 (2.79, 5.21) **	1.27 (0.91, 1.78)
Sudurpashchim province	1.14 (0.78, 1.67)	0.99 (0.67, 1.46)	0.85 (0.52, 1.39)	0.72 (0.42, 1.21)	0.82 (0.43, 1.58)	0.58 (0.30, 1.07)	1.15 (0.80, 1.63)	0.99 (0.68, 1.44)
**Residence**								
Urban	Ref	Ref	Ref		Ref		Ref	
Rural	1.02 (0.86, 1.21)		1.06 (0.85, 1.32)		1.08 (0.82, 1.43)		1.06 (0.90, 1.25)	
**Wealth Index**								
Rich	Ref	Ref	Ref	Ref	Ref	Ref	Ref	Ref
Middle	1.47 (1.14, 1.90) **	0.98 (0.76, 1.26)	1.44 (1.05, 1.99) *	0.85 (0.62, 1.16)	1.43 (0.90, 2.25)	0.97 (0.64, 1.45)	1.59 (1.25, 2.03) **	1.01 (0.79, 1.29)
Poor	1.66 (1.35, 2.05) **	1.24 (0.96, 1.60)	1.46 (1.11, 1.91) **	0.87 (0.64, 1.18)	2.30 (1.59, 3.34) **	1.61 (1.12, 2.35) *	1.70 (1.40, 2.07) **	1.18 (0.93, 1.50)
**Experience of Parental Violence**								
No	Ref	Ref	Ref	Ref	Ref	Ref	Ref	Ref
Yes	2.87 (2.32, 3.56) **	2.24 (1.82, 2.77) **	2.49 (1.91, 3.25) **	1.64 (1.27, 2.09) **	2.94 (2.13, 4.07) **	1.83 (1.37, 2.43) **	2.72 (2.20, 3.35) **	2.09 (1.69, 2.58) **
**Husband/Partner’s Education**								
Secondary or Higher	Ref	Ref	Ref	Ref	Ref	Ref	Ref	Ref
Basic	2.17 (1.76, 2.66) **	1.17 (0.94, 1.47)	1.63 (1.25, 2.12) **	0.93 (0.70, 1.23)	2.67 (1.88, 3.79) **	1.47 (1.04, 2.12) *	2.03 (1.68, 2.47) **	1.16 (0.93, 1.43)
No education	3.19 (2.46, 4.13) **	1.06 (0.79, 1.43)	3.13 (2.29, 4.28) **	1.33 (0.94, 1.89)	3.96 (2.62, 5.98) **	1.66 (1.08, 2.58) *	3.33 (2.60, 4.26) **	1.22 (0.91, 1.63)
**Husband/Partner’s Occupation**								
Professional/technical/managerial	Ref	Ref	Ref	Ref	Ref	Ref	Ref	Ref
Agriculture	1.76 (1.19, 2.62) **	0.89 (0.59, 1.35)	1.74 (1.05, 2.88) *	1.09 (0.66, 1.85)	1.98 (1.02, 3.86) *	0.94 (0.51, 1.81)	1.60 (1.12, 2.30) **	0.81 (0.55, 1.20)
Clerical/Sales/Others	1.95 (1.31, 2.89) **	1.36 (0.94, 2.00)	1.57 (0.95, 2.60)	1.11 (0.70, 1.81)	1.24 (0.61, 2.52)	0.73 (0.40, 1.37)	1.69 (1.18, 2.42) **	1.15 (0.81, 1.65)
Manual	2.49 (1.73, 3.58) **	0.94 (0.65, 1.38)	2.33 (1.47, 3.69) **	1.08 (0.68, 1.74)	2.10 (1.13, 3.90) *	0.67 (0.38, 1.24)	2.25 (1.62, 3.13) **	0.82 (0.58, 1.16)
Not working	4.36 (2.34, 8.12) **	2.72 (1.45, 5.06) **	5.54 (2.73, 11.2) **	3.82 (1.89, 7.75) **	3.04 (1.21, 7.60) *	1.10 (0.43, 2.69)	3.93 (2.18, 7.09) **	2.58 (1.40, 4.73) **
**Husband/Partner’s Alcohol consumption**								
Does not drink/Never Drunk	Ref	Ref	Ref	Ref	Ref	Ref	Ref	Ref
Is sometimes/often Drunk	3.33 (2.77, 4.01) **	2.74 (2.29, 3.28) **	3.28 (2.59, 4.15) **	2.56 (2.06, 3.20) **	3.81 (2.84, 5.12) **	2.40 (1.83, 3.16) **	3.19 (2.68, 3.81) **	2.73 (2.29, 3.25) **
**Control Behavior Displayed by Husband/Partner**								
No behavior displayed	Ref	Ref	Ref	Ref	Ref	Ref	Ref	Ref
Control behavior Displayed	5.74 (4.73, 6.98) **	4.00 (3.35, 4.78) **	10.2 (7.57, 13.7) **	7.47 (5.91, 9.51) **	11.9 (8.18, 17.3) **	7.97 (5.81, 11.1) **	6.56 (5.45, 7.91) **	4.80 (4.05, 5.71) **
**Respondent Afraid of Partner**								
Never Afraid	Ref	Ref	Ref	Ref	Ref	Ref	Ref	Ref
Sometimes or Most of the time afraid	3.55 (2.91, 4.33) **	2.29 (1.89, 2.78) **	3.65 (2.77, 4.81) **	1.94 (1.51, 2.52) **	5.98 (4.13, 8.65) **	3.26 (2.28, 4.76) **	3.39 (2.81, 4.08) **	2.03 (1.69, 2.44) **
**Education**								
Secondary or Higher	Ref	Ref	Ref	Ref	Ref	Ref	Ref	Ref
Basic	2.13 (1.68, 2.71) **	1.26 (0.98, 1.62)	1.53 (1.14, 2.06) **	0.86 (0.63, 1.17)	2.58 (1.72, 3.87) **	1.43 (0.96, 2.15)	2.00 (1.60, 2.49) **	1.29 (1.02, 1.64) *
No education	3.37 (2.66, 4.27) **	1.51 (1.10, 2.06) *	2.49 (1.87, 3.33) **	1.09 (0.76, 1.57)	3.18 (2.12, 4.77) **	1.35 (0.85, 2.15)	3.26 (2.61, 4.06) **	1.81 (1.36, 2.40) **
**Occupation**								
Professional/technical/managerial	Ref	Ref	Ref	Ref	Ref	Ref	Ref	Ref
Agriculture	2.45 (1.47, 4.07) **	1.16 (0.71, 1.95)	2.49 (1.25, 4.95) **	1.28 (0.68, 2.58)	1.56 (0.71, 3.40)	0.51 (0.25, 1.12)	2.24 (1.41, 3.55) **	1.01 (0.64, 1.63)
Clerical/Sales/Others	1.33 (0.72, 2.47)	1.21 (0.69, 2.17)	1.61 (0.71, 3.64)	1.64 (0.81, 3.50)	0.91 (0.32, 2.58)	0.81 (0.35, 1.93)	1.23 (0.70, 2.16)	1.07 (0.64, 1.83)
manual	3.42 (1.93, 6.06) **	1.83 (1.06, 3.23) *	3.16 (1.47, 6.81) **	1.83 (0.92, 3.88)	2.20 (0.90, 5.36)	0.86 (0.39, 1.98)	3.03 (1.78, 5.15) **	1.61 (0.96, 2.74)
Not working	2.10 (1.23, 3.60) **	0.95 (0.57, 1.62)	2.36 (1.15, 4.87) *	0.87 (0.45, 1.77)	1.23 (0.53, 2.86)	0.41 (0.20, 0.92) *	2.01 (1.23, 3.29) **	0.75 (0.47, 1.23)
**Internet Exposure**								
No	Ref	Ref	Ref	Ref	Ref	Ref	Ref	Ref
Yes	0.70 (0.59, 0.84) **	0.98 (0.79, 1.21)	0.80 (0.64, 1.01)	0.91 (0.71, 1.18)	0.63 (0.47, 0.85) **	0.84 (0.62, 1.14)	0.73 (0.62, 0.87) **	0.99 (0.81, 1.22)
**Media Exposure**								
Exposure to media	Ref	Ref	Ref	Ref	Ref	Ref	Ref	Ref
No exposure	1.83 (1.49, 2.24) **	1.15 (0.93, 1.42)	2.78 (2.17, 3.55) **	2.34 (1.84, 2.98) **	1.69 (1.22, 2.33) **	1.08 (0.79, 1.45)	2.05 (1.69, 2.50) **	1.36 (1.11, 1.67) **
**Ownership of Property**								
Does not own at all	Ref	Ref	Ref		Ref		Ref	Ref
Owns either alone or jointly	0.80 (0.63, 1.02)	0.80 (0.63, 1.02)	0.94 (0.70, 1.26)		0.98 (0.67, 1.43)		0.79 (0.63, 0.99) *	0.88 (0.70, 1.11)
**Participation in household decision-making**								
Participation	Ref	Ref	Ref	Ref	Ref	Ref	Ref	Ref
No participation	1.30 (1.02, 1.66) *	0.78 (0.60, 1.00)	2.00 (1.51, 2.66) **	1.24 (0.94, 1.63)	1.60 (1.10, 2.32) *	1.12 (0.79, 1.58)	1.49 (1.18, 1.88) **	0.74 (0.59, 0.94) *
**Attitude toward autonomy of sexual rights**								
Accepts sexual right	Ref	Ref	Ref	Ref	Ref	Ref	Ref	Ref
Does not accept sexual rights	2.24 (1.84, 2.73) **	1.16 (0.94, 1.43)	2.43 (1.91, 3.10) **	1.15 (0.90, 1.47)	2.49 (1.84, 3.36) **	1.41 (1.05, 1.90) *	2.45 (2.02, 2.96) **	1.23 (1.00, 1.51) *
**Attitude towards reasons for beating wife**								
Not justified	Ref	Ref	Ref		Ref		Ref	Ref
Justified for one or more reasons	1.39 (1.13, 1.72) **	1.23 (1.00, 1.52) *	1.10 (0.83, 1.47)		1.21 (0.84, 1.74)		1.36 (1.10, 1.66) **	1.21 (0.99, 1.49)

*OR* = *Odds Ratio*, *CI*: *Confidence Interval*, *Ref*: *reference group*.

*p value <0.05, ** *p-value <0.01*.

Age was significantly associated with experience of physical violence with odds being higher with the increase in age i.e., age group 25–34 (AOR: 1.76, CI: 1.37–2.29) and 35–49 (AOR: 2.13, CI: 1.58–2.87). Physical violence was also significantly associated with women’s attitude towards justifications for wife beating with those who justified it for one or more reasons being at higher odds (AOR: 1.23, CI: 1.00–1.52). Similarly, experience of emotional violence was significantly associated with exposure to media; more than two times the odds for those not exposed to any form of media (AOR: 2.34, CI: 1.842.98) compared to those who were exposed to media. Sexual violence was associated with province, with approximately half the odds of experiencing sexual violence in Lumbini province (AOR: 0.47, CI: 0.28–0.77) and Koshi province (AOR:0.58, CI: 0.36–0.93) compared to Bagmati province and women’s husband/partner who were educated up to basic level were more than one and half times more likely (AOR: 1.66, CI: 1.08–2.58) to have experienced sexual violence compared to those whose partners were educated up to secondary or higher level. Also, the likelihood of experiencing sexual violence was almost one and a half times higher among those women who did not accept autonomy of sexual rights (AOR: 1.41, CI: 1.05–1.90) than those who accepted their autonomy of sexual rights. Experience of sexual violence was also significantly associated with the type of women’s occupation with odds being lower for women not involved in any work (AOR: 0.41, CI: 0.20–0.92) compared to those that were working in professional or managerial or technical fields.

Ethnicity was significantly associated with all forms of IPVs. Women belonging to the Dalit community (AOR: 1.50, CI: 1.11–2.02), Muslim Community (AOR: 3.64, CI: 2.29–5.82), and other caste groups (AOR: 2.07, CI: 1.48–2.90), had a significantly higher likelihood of experiencing physical violence compared to women belonging to Brahmins/Chhetri community. Women belonging to the Muslim community (AOR: 1.98, CI: 1.03–3.79) and other caste groups (AOR: 1.68, CI: 1.11–2.54) were also more likely to experience sexual and emotional violence, respectively.

Wealth was significantly associated with sexual violence in the adjusted model with the poor being more likely to experience sexual violence (AOR: 1.61, CI: 1.12–2.35) compared to the rich. Witnessing parental violence was significantly associated with all forms of IPVs with the odds being more than twice (AOR: 2.24, CI: 1.82–2.77), one and a half times (AOR: 1.64, CI: 1.27–2.09), and almost twice (AOR: 1.83, CI: 1.37–2.43) more for physical violence, emotional violence and sexual violence respectively compared to those that did not witness parental violence.

Women whose husband/partner did not work were almost three times more likely to experience physical violence (AOR: 2.72, CI: 1.45–5.06) and almost four times more likely to experience emotional violence (AOR: 3.82, CI: 1.89–7.75) compared to those whose husband/partner was working in professional or managerial or technical fields. Similarly, the alcohol consumption behavior of partner/husband was also significantly associated with all forms of IPVs. Compared to those whose husband/partner did not consume alcohol or were never drunk, the likelihood of experiencing physical violence was almost three times higher (AOR: 2.74, CI: 2.29–3.28), emotional violence was two and half times higher (AOR: 2.56, CI: 2.06–3.20) and sexual violence was also almost two and half times more (AOR: 2.40, CI: 2.83–3.16) for women whose husband/partner were sometimes or often drunk. Women were also more likely to report IPVs if their husband/partner demonstrated controlling behavior. The odds were almost four times more for physical violence (AOR: 4.00, CI: 3.35–4.78), seven and half times more for emotional violence (AOR: 7.47, CI: 5.91–9.51), and about eight times more for sexual violence (AOR: 7.97, CI: 5.81–11.10) compared to those whose partner did not display any control behavior.

Similarly, women being afraid of their partners was significantly associated with all forms of IPVs. The likelihood of experiencing physical violence (AOR: 2.29, CI: 1.89–2.78), emotional violence (AOR: 1.94, CI: 1.51–2.52), and sexual violence (AOR: 3.26, CI: 2.28–4.76) was high for those that were sometimes or most of the time afraid of their husband/partner compared to those who were never afraid.

## Discussion

This study provides a comprehensive analysis of the association of individual as well as the partner/husband characteristics with IPV using the latest NDHS 2022. The study adds on to the existing literature on IPV by incorporating updated data from nationally representative sample of NDHS 2022 allowing for a more nuanced understanding of factors associated with IPV in Nepal.

This study found that 27% of women in an intimate relationship experience a form of IPV in their lifetime with the proportion of women experiencing physical violence, emotional violence, and sexual violence being 23%, 12%, and 7%, respectively. The prevalence of lifetime experience of various forms of IPV in this study was similar to NDHS 2016 [25] with a notable decrease in emotional and sexual violence as compared to the data from NDHS 2011 [26]. Various studies in Nepal report large ranges of IPV prevalence from 25% to 52% probably due to differences in methodology, sample size, and the study setting [10,27–29]. However, the prevalence of IPV could have been under-reported because of societal norms, feelings of shame, embarrassment, and the stigma associated with an open discourse on marital issues, particularly about sexual matters [12]. Also, disclosing violence perpetrated by husbands is quite difficult for women because of the culture of silence surrounding men’s acts and the normalization of violence against women [14].

Similar to few studies in low and middle-income countries, our study reported no association of age with IPV in general [30,31]. Contrary to our finding, the WHO in 2021 reported the highest rate (16%) of IPV occurred among young women aged 15 to 24 [32]. However, age was significantly associated with only physical violence. Women belonging to disadvantaged ethnic groups exhibited a higher prevalence of IPV, aligning with findings reported in other studies conducted in Nepal [10,27,33–36]. The study shows no significant relationship between wealth and IPV in general, contrary to a study among Iranian women which reported a significant relationship between socioeconomic status and all forms of violence [37]. This misalignment highlights socio-cultural dynamics reflecting deep-rooted social norms and cultural acceptance of violence transcending economic boundaries that may influence IPV differently compared to other regions. Understanding these contextual nuances is crucial for tailoring interventions that are culturally sensitive and effective in the Nepali context.

Similarly, the study showed that women with less educated or unemployed husbands were many times more likely to experience IPV. Goode’s [38] application of the resource theory by Blood and Wolfe [39] is one of the most cited articles in the literature on why IPV occurs. Goode conceptualizes violence as being like a material resource that can be used to gain obedience and compliance in the absence of material resources in the family. Violence or the threat of violence serves as a complement to material resources such as income or education. Thus, this theory leads to the expectation that less educated husbands and husbands with low socio-economic status or income are more likely to perpetrate violence against their partners [40]. In context of Nepal, where traditional gender norms and economic challenges still prevail, these findings underscore the need of policies promoting education and employment opportunities to men as well as potential strategies to reduce IPV. Low education and unemployment of husbands/ partners have been associated with the experience of IPV in other studies as well [13,41] further validating the research findings.

Drinking alcohol often or sometimes by the partner was statistically associated with all forms of IPV. This finding is supported by a study analyzing demographic and health survey data from 14 sub-Saharan African countries which reported partner’s alcohol use was associated with a significant increase in the odds of reporting IPVs in all the countries included in the analysis [42]. Some other studies also link a partner’s alcohol use with the perpetration of violence [15,43,44]. This highlights the consistent and crucial role that alcohol consumption plays in increasing the risk of IPV. Also, alcohol consumption is linked with aggressive behavior. A meta-analytic review that pooled 22 studies detected a significant overall effect and reported that male participants who consumed alcohol exhibited greater aggressive behavior against females than those who didn’t [45]. Integration of alcohol and IPV intervention/policy approaches at the population, community, relationship and individual level may provide the best opportunity for effective intervention for IPV reduction [46].

IPV was also linked with witnessing parental violence similar to another study in Nepal [13]. A study in India showed that women who witnessed their father beating their mother were more likely to accept violence [47]. Witnessing violence during childhood may result in the normalization or acceptance of violence by women [44]. Childhood experiences of violence at home reinforce normative forms of violence for both men and women which subsequently increases the likelihood of perpetration for men and acceptance for women [48]. These findings are consistently reported by various studies [49,50] hence underscoring the importance of addressing intergenerational cycles of violence in IPV interventions.

This study has shown that women belonging to lower socio-economic status families are more likely to experience sexual violence. An analysis from Demographic and Health Surveys in 36 countries also reported a higher likelihood of sexual violence among participants in poor households [51]. The study indicates an association between the attitude towards justification of wife beating with physical violence and the attitude towards the autonomy of sexual rights with sexual violence. Consistent with the application of Bandura’s social learning theory [52], women internalizing these attitudes are the results of a learned process from observations of social interactions within a cultural context where they observe that men are approved of exercising coercion and abuse to instill discipline [53]. Consistent with the findings from Kenya, a husband’s controlling nature was associated with all forms of violence [54].

Education was a protective factor only for physical violence and the risk of experiencing sexual violence was high for women in professional/managerial/technical occupations. Ownership of property and participation in household decision-making was not found to be significant. Our findings contradict the social causation perspective where increasing women’s resources such as income and education reduced both recent and longer-term probabilities of experiencing any form of IPV [55]. This study’s findings show that empowerment in terms of education and occupation alone cannot guarantee lower risks of IPV. Our finding, however, aligns with the Gender Resource theory which argues that the risk of IPV exists for empowered women as well if the husband is regressive or traditional [40]. The findings call for acknowledgment of marital and societal domains when attempting empowerment-related activities against IPV [56]. These findings indicate that a linear relationship doesn’t exist between empowerment characteristics and resources where increased resources would lead to lower risks of IPVs, but the relationship is quite complex [57]. IPV-related policies and interventions require a comprehensive approach that not only considers the socio-economic characteristics of women but also addresses societal norms and targets their husbands/partners to effectively tackle this complex issue.

### Strengths and limitations of the study

The data for this study has been pooled from a national-level survey with a multi-stage sampling procedure. Sampling weights have been used to adjust for the complex study design so that the data can be made nationally representative. Since this study captures data related to a topic that is still stigmatized in the community, respondents may have been hesitant to report their experiences of IPV, which limited our access to certain details, potentially impacting the depth of our analysis. However, this study has some limitations. First, due to the cross-sectional nature of the study, causal inference cannot be made. Second, our study solely relies on quantitative data, and we believe a mix of qualitative and quantitative data is required to help better understand IPV.

## Supporting information

S1 File Measurement of independent variables. (DOCX)
